# Interplay between STAT3, Cell Adhesion Molecules and Angiogenesis-Related Parameters in Gastric Carcinoma. Does STAT3 Really Have a Prognostic Value?

**DOI:** 10.3390/medicina55060300

**Published:** 2019-06-23

**Authors:** Miljan Krstić, Nikola M. Stojanović, Slavica Stojnev, Goran Radenković, Jovana Čukuranović Kokoris, Bojan Mladenović, Ljubinka Janković Veličković

**Affiliations:** 1 Department of Pathology, Faculty of Medicine, University of Niš, 18000 Niš, Serbia; slavicastojnev@gmail.com (S.S.); dravel@open.telekom.rs (L.J.V.); 2 Center for Pathology, Clinical Center Niš, 18000 Niš, Serbia; 3 Faculty of Medicine, University of Niš, 18000 Niš, Serbia; nikola.st90@yahoo.com; 4 Department of Histology and Embryology, Faculty of Medicine, University of Niš, 18000 Niš, Serbia; radenkog@gmail.com; 5 Department of Anatomy, Faculty of Medicine, University of Niš, 18000 Niš, Serbia; jovana.c85@gmail.com; 6 Department of Internal Medicine, Faculty of Medicine, University of Niš, 18000 Niš, Serbia; bovail@gmail.com; 7 Clinic of Internal Medicine, Clinical Center Niš, 18000 Niš, Serbia

**Keywords:** gastric cancer, STAT3, cancer aggressiveness, angiogenesis, cell adhesion

## Abstract

*Background and objectives:* Gastric cancer (GC) is one of the deadliest malignancies, with the underlying pathophysiological mechanisms still not completely understood. In this study, we aimed to investigate the signal transducer and activator of transcription 3 (STAT3) moleculeconnection with the pathological features of GCs, and the expression of cell adhesive molecules (E-cadherin and β-catenin) and angiogenesis-related factors (vascular endothelial growth factor (VEGF), HIF1α, and CD31)). *Materials and Methods:* This study comprised 136 cases of GCs with data related to the patients’ demographic characteristics (age, gender) and pathological features (tumor location, gross type, Laurens’ type of GC, histological differentiation, invasion depth, lymphovascular invasion and the presence of metastases) which were correlated with STAT3 expression. Additionally, STAT3 expression and the expression of adhesive molecules and angiogenesis-related factors were studied by immunohistochemical methods. *Results:* The expression of STAT3 was found to be significantly associated with the occurrence of poorly differentiated GCs in the lower portion of the stomach and with the presence of distant metastases. Interestingly, none of the investigated parameters related to cell adhesion or to angiogenesis were found to be related to the expression of STAT3. *Conclusions:* The lack of significant differences between the studied STAT3 expression and some of the molecules associated with different cancer features might be due to the characteristics of the studied population sample associated with the origin, heterogeneity, and cancer pathophysiological background. Nonetheless, the results of our study suggest that STAT3 could be a useful marker for the presence of distant GC metastases, which further indicates that STAT3 action might involve some other signaling molecules/pathways that warrant further elucidation.

## 1. Introduction

Despite the steady declining incidence, with over one million newly diagnosed cases each year, gastric cancer (GC) continues to be one of the most frequent and one of the deadliest malignancies that can be encountered in human pathology [1]. Gastric cancers are histologically classified into two main histological types—intestinal, formed of glands with various degree of differentiation, and diffuse type, consisting of discohesive tumor cells [2,3]. These two histological types are the result of distinct pathogenetic pathways and display different biological and etiological characteristics [3]. Significant geographic and epidemiologic differences in GC worldwide distribution are associated with a strong environmental impact on the tumor etiopathogenesis [4]. The role of chronic inflammation in carcinogenesis has been previously established, and the infection with *Helicobacter pylori* remains the most important risk factor [5]. A recent study suggested that the diet with inflammatory potential significantly contributes to the risk of GC in the European adult population [6]. Inflammation inappropriately activates many intracellular signaling pathways and causes dysregulation of intracellular signaling pathways that underlies molecular mechanisms in carcinogenesis. Recently, the aberrations in Janus-activated kinase (Jak)/ Signal transducer and activator of transcription (STAT) signaling pathway have been recognized as an important factor in the development and progression of GC [7]. The cascade involving Jak/STAT is essential in transfer of cytokine and growth factor signals, in the regulation of cell proliferation, differentiation, migration, and apoptosis. Signal transducer and activator of transcription 3 (STAT3), the prominent member of STAT family, is involved in gastric carcinogenesis in several different and diverse directions. 

The pivotal role of STAT3 in the regulation of epithelial-to-mesenchymal transition (EMT) in GC, a fundamental process that enables cancer cells to obtain mesenchymal properties, including loss of contact inhibition and increased motility, allowing them to disseminate, has been previously revealed [8]. EMT is inherently associated with the loss of intercellular adhesion, which is one of the major hallmarks of tumor progression, invasiveness, and metastatic spread. Cell adhesion largely depends on cadherins, located in the cell membrane, which are frequently found altered in diffuse type of GC [9]. The loss of E-cadherin expression, which may be caused by genetic alterations or epigenetic mechanisms, is an early event in gastric carcinogenesis, suggesting the tumor-suppressive properties of E-cadherin. The expression of E-cadherin is modified by STAT3 via regulation of Snail, the master transcription factor in EMT. This membrane protein maintains epithelial phenotype through its bond to β-catenin, the effector molecule of the WNT/β-catenin pathway, which regulates cell cycle homeostasis and cell migration in the gastric epithelium [10]. It was found that the loss of regular membranous/cytoplasmic expression of β-catenin in diffuse and mixed-type GC significantly correlates with pathologic stage [11].

The supportive influence of STAT3 to tumor angiogenesis was described in several malignancies [12]. In response to the microenvironmental factors, triggered by inflammation or hypoxia, STAT3 signaling regulates the activity of HIF1α, the central switch of tumor angiogenesis. In turn, HIF1α stimulates the transcription of genes involved in new blood vessels formation. In addition, it has been found that STAT3 increases the expression of vascular endothelial growth factor (VEGF) directly, through binding to its promoter. The vascular endothelial growth factor is a family of growth factors with immediate roles in angiogenesis in physiological state, as well as in malignant disease. GC is associated with increased angiogenesis, where the pronounced expression of VEGF correlates well with microvessel density (MVD), a micromorphological parameter with prognostic significance in various cancers [13]. Moreover, several anti-angiogenic therapeutics havebeen implemented in the clinical treatment of numerous neoplasms, which emphasizes the significance of the precise determination of the angiogenic profile of the tumor.

Based on the assumptions that there is a connection between STAT3 and EMT and/or angiogenesis signaling pathways in GC, this study aimed to investigate the possible relations between immunohistochemical expression of STAT3 and adhesive molecules (E-cadherin and β-catenin) and angiogenesis-related factors (VEGF, HIF1α and MVD). Moreover, we will assess the connection between the expression of STAT3 and some of the pathological features associated with GCs.

## 2. Materials and Methods

### 2.1. Patients and Clinicopathological Characteristics

This study comprised 136 formalin-fixed, paraffin-embedded GC specimens, obtained from patients who had undergone total or partial resection of the stomach at the Department of general surgery, Clinical Center Niš. The cases of GC were diagnosed at the Center of Pathology, Clinical Center Niš during the 5-year period (2012–2016). All tumors were divided into two groups regarding the localization of the main tumor mass into the upper portion of the stomach (subcardial and corporal location) and a lower portion (antropyloric) of the stomach. Tumors of the gastric cardia and gastro/esophageal junction were excluded from the study. Cancer tissue sections stained with hematoxylin and eosin (HE) were used for precise histologic diagnosis and the assessment of pathological features/parameters. Pathologic classification of GC was performed according to the 2010 World Health Organization classification [14], while the staging was performed according to the Eight edition of UICC TNM Classification [15]. The study was approved by the Ethics Committee of the Clinical Center Niš, Serbia (Decision No 19869/15).

### 2.2. Immunohistochemical Analysis and Scoring

Immunohistochemical analysis was performed on representative sections of GC tissue samples using the following primary antibodies: a mouse monoclonal antibody to STAT3 (124H6, Cell Signaling Technology, 1:300 dilution), a rabbit polyclonal antibody to VEGF (A-20, sc-125, Santa Cruz Biotechnology, 1:200 dilution), a rabbit monoclonal antibody to HIF1α, (H-206: sc-10790, Santa Cruz Biotechnology, 1:250 dilution), and mouse monoclonal antibodies against CD31 (Clone JC/70A, Abcam, 1:100 dilution), E-cadherin (clone 36, BD Biosciences, 1:100 dilution), and β-catenin (clone 14, BD Biosciences, 1:250 dilution). In brief, 3 μm thick tissue sections were deparaffinized in xylene and rehydrated in a graded series of ethanol, and deionized water. Following adequate heat-induced antigen retrieval procedure, the endogenous peroxidase activity was quenched, and the slides were rinsed thoroughly with phosphate buffered saline. The primary antibodies were applied and the slides were incubated in a water bath for one hour at room temperature. Appropriate positive and negative controls were included in every immunostaining procedure. For the reaction visualization, a standard immunoperoxidase detection system was applied according to the manufacturer’s instructions (DAKO LSAB2R system-HRP, Dako, Danmark), and diaminobenzidine was used as a chromogen. Slides were afterward counterstained with Mayer’s hematoxylin, dehydrated, and mounted.

Stained slides were reviewed independently by three experienced pathologists (MK, SS, LJV) and any interobserver discrepancies were resolved using a multiple-headed microscope. Positive immunohistochemical reaction, the presence of investigated protein, was verified in a form of brown staining of cell membranes, cytoplasm, or cell nuclei. For STAT3 immunoexpression both nuclear and cytoplasmic staining was observed. Nuclear expression was considered relevant for HIF1α, nuclear, and cytoplasmic for VEGF, while CD31, E-cadherin and β-catenin stained cell membranes, and perimembranous cytoplasmic rim. For the purposes of statistical analysis, immunohistochemical staining results for all markers were placed in one of the two categories, high or low, while for the cell adhesion molecules, the categories were normal (retained diffuse membranous staining pattern), or altered (heterogeneous, decreased, or completely lost staining for E-cadherin and β-catenin). For STAT3, HIF1α, and VEGF immunohistochemical reaction was scored as follows: low if ≤10% of cells were stained, and high if ≥10% were stained, with at least intermediate or strong staining intensity, as previously described [16,17].

Microvessel density (MVD) was determined based on the identification of endothelial cells by immunostaining to CD31, with the help of digital software ImageJ. For the calculation of MVD, active areas of angiogenesis, so-called hot spots, were selected on low power slide scanning. For each case, images from ten high power fields (400×) in the hot spot areas were recorded. Every single endothelial cell stained for CD31 found solitary in the tumor stroma, isolated from adjacent vessels with visible lumens, was counted separately and considered to represent a countable microvessel. The MVD was expressed as the number of blood vessels per 1 mm^2^, and based on the average value all cases were divided into two groups, the one with low or high MVD [18].

### 2.3. Statistical Analysis

Collected data are presented as frequency distributions expressed as percentages or median values and are given in Table 1 and Table 2. All statistical comparison was done in Statistical Package for Social Sciences, version 21.0 statistical software (SPSS, IBM Corp, 2012). Categorical variables are analyzed using either Chi-square or Fisher’s exact test with Yates correction depending on the number of cases per cell. The probability (*p*) values less or equal to 0.05 were considered to be statistically significant. Additionally, in some cases, the adjusted residual was calculated for each cell and its values were considered to be statistically significant when they were ≥2 or ≤−2.

## 3. Results

Our study sample comprised of 136 cases of different GC, where 96 (70.6%) of patients were males and the remaining 40 (29.4%) were females, with a mean age of 64.9. High immunohistochemical expression of STAT3 was detected in around one third (31.6%) of all studied GCs (Table 1).

**Figure 1:**
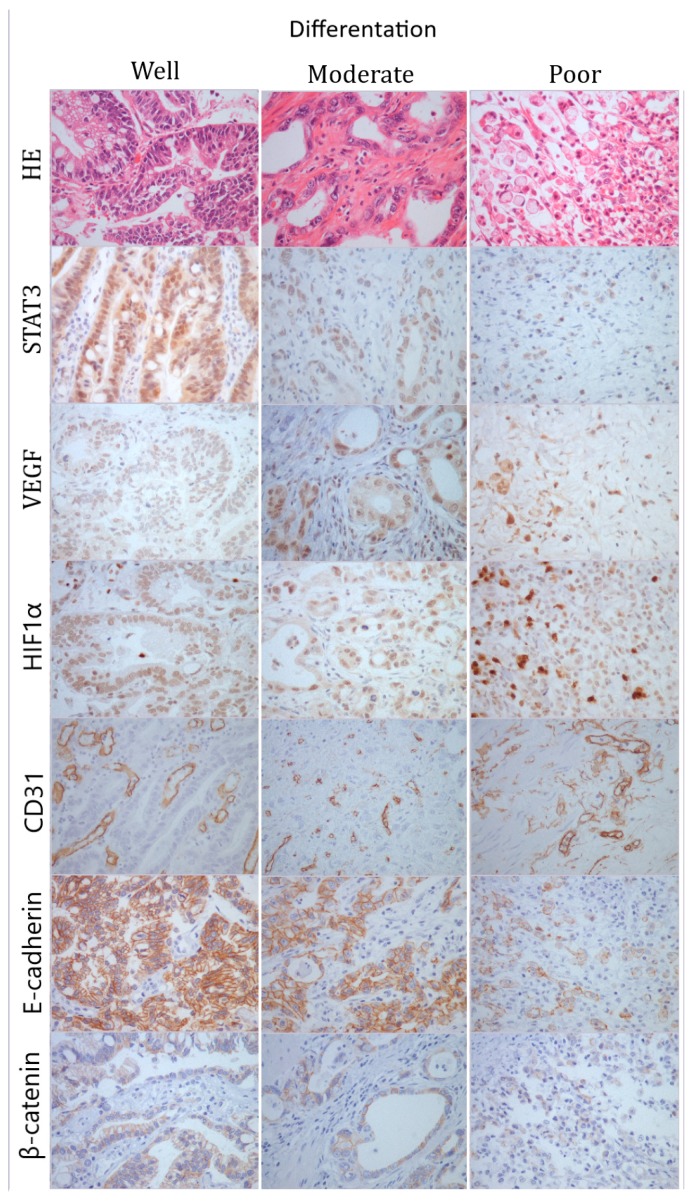
Representative photomicrographs of HE and immunohistochemical staining to STAT3, VEGF, HIF1α, CD31, E-cadherin, and β-catenin in gastric cancer; first column–well-differentiated tumor; second column–moderately differentiated tumor; third column–poorly differentiated tumor. Original magnification ×400.

Statistical analysis of demographic data and pathological features of studied GC revealed that high STAT3 expression is significantly associated with GC predominantly located in the lower portion of the stomach (antrum and pylorus location) that have poor histological differentiation (*p*=0.012, z value ≥2), and give distant metastases (Table 1). On the other hand, low STAT3 expressing GCs are found almost equally in both upper and lower portion of the stomach, but have significantly less poor histological differentiation (*p*=0.012, z value ≤−2), and less distant metastasis (Table 1). The expression of STAT3 was not found to be statistically significantly connected with any other studied parameter (patients’ age, GC gross type, Laurens’ classification of GC, invasion depth, vessel, and lymph node invasion; Table 1). The representative immunohistochemical stainings are shown in Figure 1.

None of the investigated immunohistochemical parameters associated with neovascularization and cell adhesion were found to be connected with the expression of STAT3 (*p*>0.05; Table 2). Tumors with high VEGF expression showed more frequently high immunoreactivity to STAT3 compared to VEGF low GCs (39.0% vs. 26.0%); however, this difference was not statistically significant.

## 4. Discussion

In evaluation of GC aggressiveness there are numerous pathological features that should be taken into account, including histological differentiation, tumor depth, presence of metastases, as parameters with well-established biological significance. In addition, the expression of certain molecules can provide a better insight into tumor aggressiveness through the assessment of cancer tissue vascularization, cells’ potential to divide, undergo apoptosis, or acquire the potential to spread.

The tumor localization represents an important feature of GC that is in direct connection with the type of surgery and further with the patient’s post-surgery recovery [19]. One of the previous publications suggested that the expression of STAT3 in its phosphorylated form is not related to tumor localization [20], which is contrary to the results of our study. On the other hand, GC that are related to *H. pylori* infection and consequential inflammation, stimuli for STAT3 expression, are found to be localized in the antral (lower) portion of the stomach [7,21]. The results of our study indeed showed that highly expressing STAT3 GCs are predominantly located in the lower portions of the stomach.

The presence of poor histological differentiation within GC has been associated with high STAT3 expression according to the findings of our study (Table 1). Up to now, there is no clear attitude towards such findings, where some authors report the connection between STAT3 expression and poor histological differentiation [22,23], while others did not notice such a connection [24]. The results of our study agree with the present knowledge connected to the STAT3 signaling pathway which, when activated, leads to the modulation of cell growth, proliferation, and differentiation [22].

Late diagnosis of GC is highly frequently associated with the presence of distant metastases, when the chance of complete remission and survival more than 18 months is drastically decreased [25]. In our studied GC sample high STAT3 expression was found to be linked with the distant metastases (Table 1), which has not been previously precisely elaborated. Some studies dealing with the role of STAT3 in GCs even excluded patients with distant metastases [26] or chosen their sample from a larger sample [16,17], thus limiting the pool of studied GCs. The occurrence of distant metastases is in direct connection with the formation of new blood vessels and EMT which is associated with the loss of cell adherence molecules [27,28]. Such statically significant connection between the STAT3 expression and the presence of distant metastases in the studied sample prompted us to further pursue the potential connections between STAT3 and neovascularization and/or cell adhesion molecules expression in GCs.

Formation of new blood vessels within the tumorous mass represents an important characteristic of tumors that enables tumor tissue growth and raises the possibility of cancerous cell spread through the bloodstream [29]. Although our results did not reveal the statistically significant association between STAT3 expression and herein studied neovascularization immunohistochemical markers (VEGF, HIF1α and MVD), previous studies have suggested otherwise [26,28]. Thus, these results might seem somewhat controversial since it is proven that STAT3, when stimulated by the inflammatory cytokines, acts as direct transcriptional activator for different important regulatory proteins, including VEGF [26]. Furthermore, there are publications suggesting that the expression of VEGF is dependent on the activation of HIF1α [30] and directly manifests with an increase in MVD [16]. The lack of significant connection between STAT3 and MVD might be associated with an inadequate marker selected for MVD measurement since it is still debatable what might be the appropriate marker that truly reflects MVD [16,29]. Also, STAT3 and VEGF co-expression was found to be associated with lymph node metastasis [28]; however, in our study such clear connection was not detected, although in high STAT3 expression GC patients the occurrence of lymph node metastasis was slightly higher than in those with low expression (76.7 vs. 61.3%, respectively; Table 1).

Our results suggest that STAT3 expression is significantly associated with poor tumor histological differentiation and the presence of distant metastases, the two features of GC previously shown to be tightly related to pronounced EMT [31]. In the present study, we used the two most common, and among the most promising immunohistochemical markers for EMT; however, molecular pathways involved in EMT are numerous and often intersecting, and converge to EMT execution phase, where E-cadherin expression is lost, thus there are certainly numerous other molecules that could reflect the process of EMT in GC cells [32]. The fact that there is no statistical relationship between E-cadherin loss and STAT3 expression does not exclude disarrangements in proximal arms of EMT regulating pathways. Further investigational efforts on STAT3 roles in GC might involve other important EMT–associated molecules (TGF-β, Smad proteins, Snail, Zeb1, N-cadherin).

The connection between signaling pathways associated with neovascularization and loss of cell adhesion (EMT) are partially intertwined [29]. Specifically, the same inflammatory signal that triggers STAT3 also causes an increase in matrix metalloproteinase (MMP) activities that are responsible for the cancerous tissue microenvironment reorganization, as well as for the formation of new blood vessels [33]. However, the carcinogenesis of GC is very perplexed and probably involves many other intrinsic and environmental influences than exclusively inflammatory signals. Contributing to such a hypothesis are the results of some previous studies where the expression of STAT3 was not found to be connected to *H*. *pylori* infection [17].

The detected discrepancies in the obtained results may lay in the nature of the investigated samples. The studies suggesting the importance of STAT3 as a marker of tumor progression and aggressiveness are based on GC samples derived from an Asian population, while the ones used in this study are from Caucasian (European) patients. Also, numerous studies narrowed the sample by applying different inclusion/exclusion criteria which resulted in a more or less compact sample. On the other hand, we conducted our survey on every subsequently diagnosed GC in a five-year period that have been examined in our institution, which makes our study sample quite heterogeneous.

## 5. Conclusions

The present study conducted on 136 cases of GC diagnosed in patients originating from a relatively small population (around 1,000,000) of Southeastern Serbia. Statistical analysis revealed that the expression of STAT3 signaling molecule is connected with tumor location, histological differentiation, and the presence of distant metastases. However, we failed to detect any significant connection between STAT3 and neovascularization (VEGF, HIF-1α and microvascular density) and cell adhesion molecules (E-cadherin and β-catenin) expression. The lack of significant difference between the studied STAT3 expression and some of the molecules associated with different cancer features might be due to studied sample characteristics that could involve origin, heterogeneity, and cancer pathophysiological background. Nevertheless, the results of our study suggest that STAT3 could be a useful marker for the presence of distant GC metastases, which further indicates that STAT3 action might involve some other signaling molecules/pathways that warrant further elucidation.

## Figures and Tables

**Table 1:** Demographic data and pathological features associated with STAT3 expression.

Characteristics		STAT3 Expression		*p*-Value
		Low	High	
**Number of cases (*n*)**		93	43	-
Male gender (*n*, %)		63, 67.7	34, 79.1	0.220
Age				
	Median (IQR)	64 (64–64)	65(65–65)	0.492
	˂60 years old	27, 29	8, 18.6	0.214
Tumor location (*n*, %)				
	Upper portion	44, 47.3	10, 23.3	0.009
	Lower portion	49, 52.7	33, 76.7	
Gross type (*n*, %)				
	Elevated	25, 26.9	13,30.2	0.268
	Flat	39, 41.9	12, 27.9	
	Depressed	29, 31.2	18, 41.9	
Laurens’ classification (*n*, %)				
	Intestinal	65, 69.9	22, 51.2	0.054
	Diffuse	28, 30.1	21, 48.8	
Histological differentiation (*n*, %)				
	Well	8, 8.6	0, 0	0.012
	Moderate	51, 54.8	17, 39.5	
	Poor	34, 36.6 ^b^	26, 60.5 ^a^	
Invasion depth (*n*, %)				
	T1	2, 2.2	2, 4.7	0.709
	T2	38, 40.9	14, 32.6	
	T3	39, 41.9	19, 44.8	
	T4	14, 15.1	8, 18.6	
Lymphovascular invasion				
	Present	42, 45.2	25, 58.1	0.197
Lymph node metastasis (*n*, %)				
	Present	57, 61.3	33, 76.7	0.083
Distant metastasis (*n*, %)				
	Present	4, 4.3	8, 18.6	0.018

^a^ adjusted residual ≥2; ^b^ adjusted residual ≤−2.

**Table 2:** Association of immunohistochemical markers and STAT3 expression.

Characteristics		STAT3 Expression		*p*-Value
		Low	High	
**Angiogenesis associated parameters**				
VEGF (*n*, %)				
	Low	57, 61.3	20, 46.5	0.137
	High	36, 38.7	23, 53.5	
HIF1α (*n*, %)				
	Low	43, 46.2	18, 41.9	0.712
	High	50, 53.8	25, 51.8	
MVD (number/mm^2^)				
	Low	52, 55.9	21, 48.8	0.465
	High	41, 44.1	22, 51.2	
Cell adhesion parameters				
E-cadherin (*n*, %)				
	Normal	50, 53.8	22, 51.2	0.854
	Altered	43, 46.2	21, 48.8	
β-catenin (*n*, %)				
	Normal	53, 57.6	25, 58.1	0.553
	Altered	39, 42.4	18, 41.9	
